# Recent Developments in the Methods and Applications of Electrostatic Theory

**DOI:** 10.1021/acs.accounts.3c00068

**Published:** 2023-08-16

**Authors:** Elena Besley

**Affiliations:** School of Chemistry, University of Nottingham, University Park NG2 7RD, U.K.

## Abstract

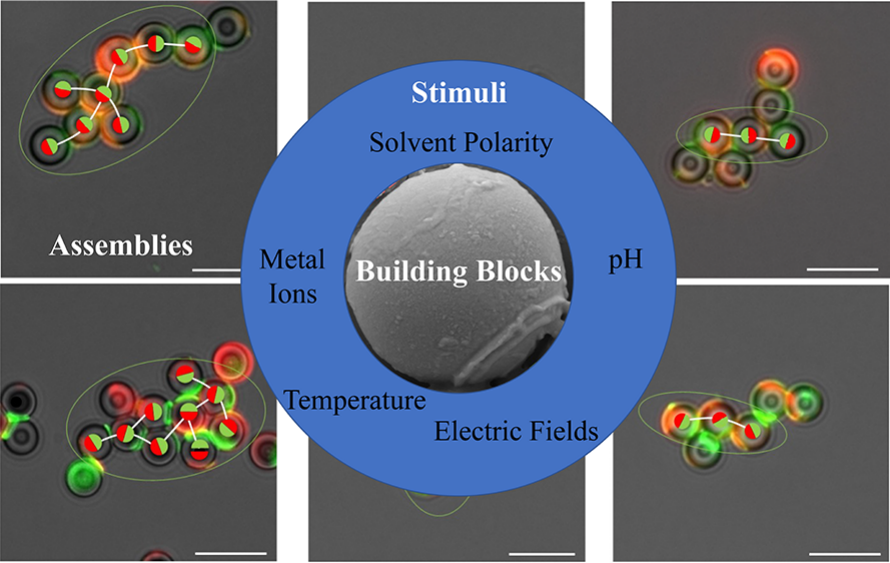

The review improves our understanding of how electrostatic interactions in the electrolyte, gas phase, and on surfaces can drive the fragmentation and assembly of particles. This is achieved through the overview of our advanced theoretical and computational modeling toolbox suitable for interpretation of experimental observations and discovery of novel, tunable assemblies and architectures. In the past decade, we have produced a significant, fundamental body of work on the development of comprehensive theories based on a rigorous mathematical foundation. These solutions are capable of accurate predictions of electrostatic interactions between dielectric particles of arbitrary size, anisotropy, composition, and charge, interacting in solvents, ionized medium, and on surfaces. We have applied the developed electrostatic approaches to describe physical and chemical phenomena in dusty plasma and planetary environments, in Coulomb fission and electrospray ionization processes, and in soft matter, including a counterintuitive but widespread attraction between like-charged particles.

Despite its long history, the search for accurate methods to provide a deeper understanding of electrostatic interactions remains a subject of significant interest, as manifested by a constant stream of theoretical and experimental publications. While major international effort in this area has focused predominantly on the computational modeling of biocatalytic and biochemical performance, we have expanded the boundaries of accuracy, generality, and applicability of underlying theories. Simple solvation models, often used in calculating the electrostatic component of molecular solvation energy and polarization effects of solvent, rarely go beyond the induced dipole approximation because of computational costs. These approximations are generally adequate at larger separation distances; however, as particles approach the touching point, more advanced charged-induced multipolar descriptions of the electrostatic interactions are required to describe accurately a collective behavior of polarizable neutral and charged particles. At short separations, the electrostatic forces involving polarizable dielectric and conducting particles become nonadditive which necessitates further developments of quantitatively accurate many-body approaches. In applications, the electrostatic response of materials is commonly controlled by externally applied electric fields, an additional complex many-body problem that we have addressed most recently, both theoretically and numerically.

This review reports on the most significant results and conclusions underpinning these recent advances in electrostatic theory and its applications. We first discuss the limitations of classical approaches to interpreting electrostatic phenomena in electrolytes and complex plasmas, leading to an extended analytical theory suitable for accurate estimation of the electrostatic forces in a dilute solution of a strong electrolyte. We then introduce the concept and numerical realization of many-body electrostatic theory focusing on its performance in selected experimental cases. These experiments underpin, among other applications, electrostatic self-assembly of two-dimensional lattice structures, melting of ionic colloidal crystals in an external electric field, and coalescence of charged clusters.

## Key References

- BichoutskaiaE.; BoatwrightA. L.; KhachatourianA.; StaceA. J.Electrostatic analysis of the interactions between charged particles of dielectric materials., Journal of Chemical Physics2010, 133, 0241052063274610.1063/1.3457157.^1^*A fundamental solution to the problem of calculating electrostatic interactions between dielectric particles of arbitrary size, charge, and separation distance. The theory predicts experimental conditions where particles with the same sign of charge are strongly attracted to one another.*
- DerbenevI. N.; FilippovA. V.; StaceA. J.; BesleyE.Electrostatic interactions between charged dielectric particles in an electrolyte solution. Journal of Chemical Physics2016, 145, 0841032758690010.1063/1.4961091.^2^*Theory is developed to address a significant problem of how charged particles interact in a dilute solution of a strong electrolyte. The proposed solution advances the celebrated Derjaguin–Landau–Verwey–Overbeek (DLVO) theory.*
- LindgrenE. B.; StammB.; MadayY.; BesleyE.; StaceA. J.Dynamic simulations of many-body electrostatic self-assembly. Philosophical Transactions of the Royal Society A2018, 376, 2017014310.1098/rsta.2017.0143PMC580591329431686.^3^*Experimental studies relating to electrostatic self-assembly have been the subject of dynamic computer simulations to reveal how particle polarizability can influence the assembly process.*
- HassanM.; WilliamsonC.; BaptisteJ.; BraunS.; StaceA. J.; StammB.; BesleyE.Manipulating particle interactions with electric fields and point charges: A general electrostatic many-body framework. Journal of Chemical Theory and Computation2022, 18, 6281–62963607505110.1021/acs.jctc.2c00008PMC9558380.^4^*This work yields a rigorous analytical formalism and an efficient numerical method for the quantitative evaluation of the electrostatic interactions between dielectric particles in an external electric field. This formalism also allows for inhomogeneous surface charge distributions.*

## Introduction

The search for accurate methods providing a general description and deeper understanding of electrostatic interactions in the gas phase, in solution, and on surfaces remains a subject of intense interest.^1−5^ Gas phase experiments where charged particles conform to the Rayleigh instability relationship, such as Coulomb fission of multiply charged clusters and the production of multiply charged ions through electron impact ionization,^6−9^ can be readily modeled using electrostatic theory describing the interaction between two charged dielectric particles in vacuum.^1^ This theory takes into account charge induced surface polarization, i.e. an instantaneous redistribution of surface charge on a particle caused by the presence of external electric charge. This applies to both particles and results in a static configuration that leads to overall repulsive or attractive interactions. The attraction between like-charged particles at close separation distances is a particularly interesting phenomenon which is strongly influenced by the polarization effects.^1,10^

The two-body formalism^1^ reproduced closely the measurements of delayed Coulomb fission of size-selected dication clusters comprising water, ammonia, acetonitrile, pyridine, benzene;^7^ triply and/or quadruply charged molecular clusters of benzene, acetonitrile, and tetrahydrofuran;^8^ and experiments on the stability of multiply charged fullerenes as well as carbon and fullerene clusters.^6^ This success in interpreting the Coulomb fission near the Rayleigh instability limit makes the two-body solution^1^ a practical alternative to modeling the kinetic energy release based on the assumption of a uniform distribution of surface charge.^6−8^ The electrostatic framework^1^ has been also applied to study aerosol growth in the atmosphere of Titan^11^ and the coalescence of ice and dust particles in the mesosphere and lower thermosphere of Earth.^12^

Useful general expansions of the two-body theory^1^ have been developed, with particular focus on achieving a reliable numerical convergence of the analytical expressions describing the electrostatic energy and force acting between the particles. These cover a simple extension to include an isotropic and uniform dielectric medium which offers a quantitative agreement with experimental measurements of the electrostatic force between charged microparticles suspended in nonpolar solvents, such as poly methyl methacrylate (PMMA) spheres suspended in hexadecane.^13^ The localized surface (point) charges have been also incorporated^9^ to describe the electrostatic interaction between particles with nonuniform surface charge. Other extensions of the two-body problem include models describing electrostatic interactions on a surface^14^ and between spheroidal dielectric particles.^15^ The particle–plane model^14^ has been applied to describe the interactions between a charged lactose sphere and a neutral glass surface, and between a charged polystyrene sphere and a neutral graphite surface.^14^ In both experiments, a charged particle was found to be attracted to a neutral support. The theory confirmed that the attractive force was mainly of the electrostatic polarization origin and it extended to a longer range of sphere–plane separations than previously reported.

The collective behavior and nonadditive nature of the forces acting on charged polarizable particles in a cluster or lattice necessitates the application of a many-body theory^3^ capable of describing the long-range nature of the Coulomb interactions and many-body polarization effects. Hence, advanced charged-induced multipolar solutions to the electrostatic interactions are required to describe any collection of polarizable charged (and neutral) particles.^4,16^ In order to reduce computational costs, solvation models typically use the simple induced dipole approximation, which is often sufficient at long separations. However, as particles approach one another a very significant number of multipolar polarization terms is required for the energy to converge to its correct value.^17^ For many-body electrostatic problems, quantitatively accurate predictions require particularly efficient and nontrivial numerical solutions.^4,16^ A many-body electrostatic method with linear scaling of the approximate solution with respect to the number of particles has recently been demonstrated.^4^ It also has an additional capability of representing local surface charges as point charges or as patches through the description of nonuniform surface charge density.^4^

These major advancements in electrostatic theory^1,2,14,18^ and their numerical realizations^4,16^ explain how these interactions can influence the assembly of particles into structured functional materials, ultimately leading to the discoveries of novel, tunable, hierarchically structured assemblies and architectures. Particles may possess geometric, interfacial or compositional anisotropy, as found in nonspherical and patchy particles,^13^ colloidal solutions^19^ and superlattices.^20,21^ Assembly of anisotropic colloids into hierarchically ordered, reconfigurable architectures provides a basis for the design of exotic new materials and controllable optical and imaging devices in emerging technologies. Our current capabilities to produce such materials are limited by fundamental problems with control and optimization of the assembly processes. However, a steady increase in the number of experimental papers investigating spontaneous or directed electrostatic (self-) assembly indicates significant interest in this field.

## Electrostatic Interactions in an Electrolyte Solution

### Beyond the DLVO Approximation

Electrostatic interactions between charged particles in a medium govern many important physical and chemical phenomena in colloidal science,^22^ complex plasmas,^23^ biological systems,^24^ and atmospheric processes.^25^ Traditionally, our understanding of these interactions rely on the well-known Derjaguin–Landau–Verwey–Overbeek (DLVO) approximation^26,27^ and its variations, all of which assume that polarization effects can be neglected. In ionic atmospheres, solutions to the well-known Poisson–Boltzmann (PB) equation are held as a gold standard. While certainly useful for providing physical insights, the PB equation violates a general reciprocity principle, and we can not expect the ionic medium to be additive.

If the surface electrostatic potential is relatively low, the electrostatic force between two small ions can be defined by the Debye–Hückel approximation
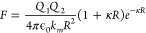
1where κ^–1^ is the Debye length, *R* is the separation distance between two ions represented by point charges *Q*_1_ and *Q*_2_, *k*_*m*_ is the dielectric constant of the medium, and ϵ_0_ is the dielectric permittivity of vacuum. A number of attempts have been made at extending the original Debye–Hückel theory of dilute electrolytes^28^ to account for polarization effects and a finite particle size (note, the DLVO approximation only deals with point charges). Notably, in the late 1930s Levine^29^ described the interaction between two identical colloidal particles with a uniform surface charge up to the quadrupole term. Almost 35 years later,^30^ he extended his solution using an infinite multipolar expansion^30^ and included the finite region of an ionised medium. However, the latter solution turned out to be dependent on the choice of boundary conditions. Separate efforts have been made in this field to understand the screening effects of concentrated electrolyte solutions which require the development of nonlocal electrostatics models in order to describe properly a fine balance between the local packing effects and the long-range Coulomb interaction.^31,32^

In 2016, Derbenev et al.^2^ developed an extended analytical solution to address the problem of two charged dielectric particles interacting in a dilute solution of strong electrolyte. This methodology is based on an infinite multipolar expansion of the electric potential, electrostatic force, and surface charge density in terms of Legendre polynomials, and it accurately captures polarization effects on the surface of the particles and in the medium. The polarization model^2^ requires a small number of input parameters such as charge, radius, and dielectric constant of the particles and the permittivity and the Debye length of the medium. The derived solution describes weak screening at large interparticle separations, which typically corresponds to the interactions of small ions with a constant charge. If only the monopole and dipole terms are considered in the analytical expansion of the electrostatic force, then the solution agrees exactly with the classical analytical expressions for ion–ion and ion–molecular interactions in a medium. The methodology^2^ has been validated against experimental measurements^33^ for two poly methyl methacrylate (PMMA) spheres in a nonpolar solvent, hexadecane. It also allowed us to assess quantitatively errors inherent in the DLVO-based approximations and to show that these approximations do not provide sufficient accuracy at short separation distances, especially with increasing asymmetry in charge and/or size of interacting particles and magnitude or placement of the charges.

**Figure 1:**
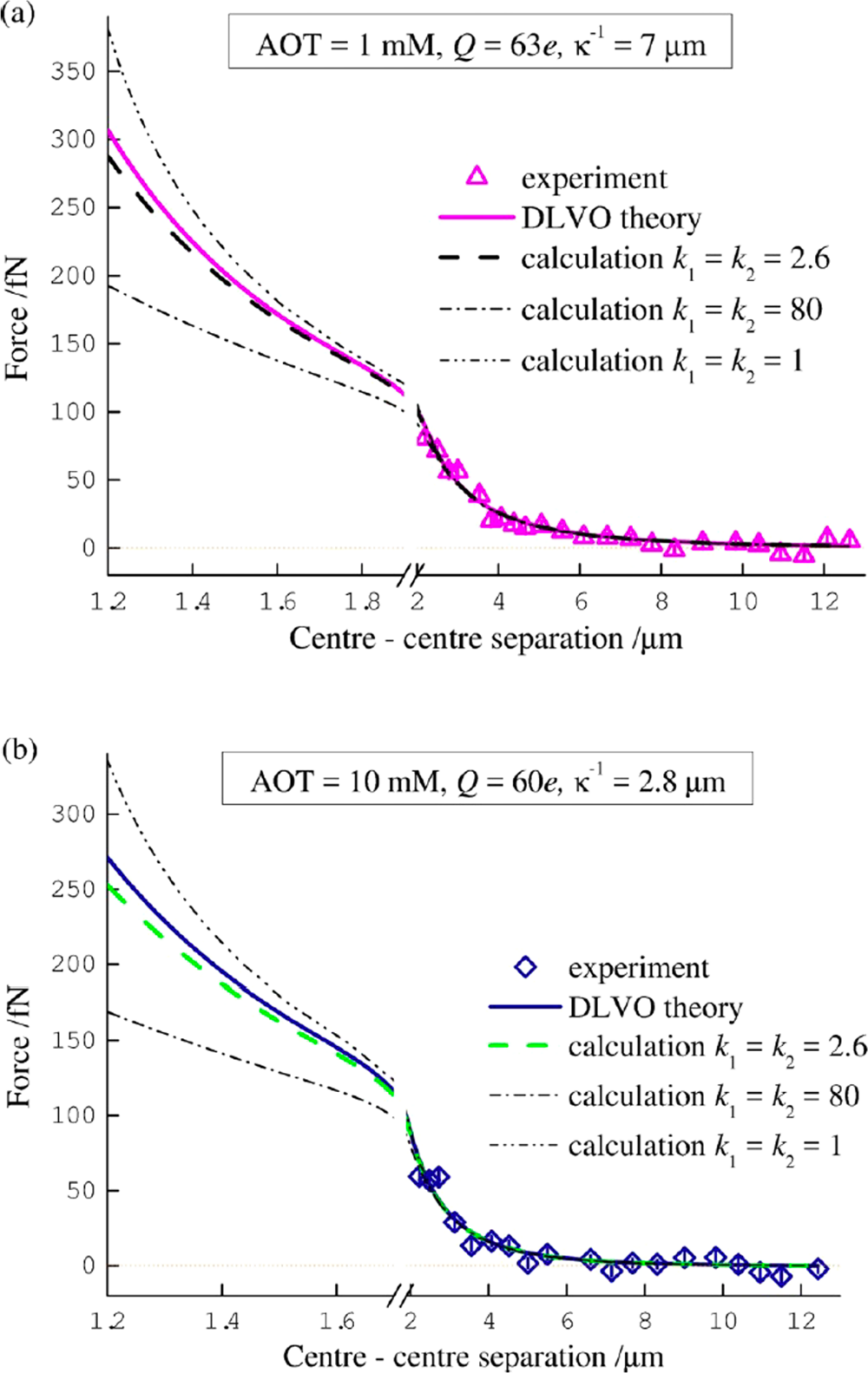
Experimental measurements,^33^ shown as diamonds, for the electrostatic force between two charged PMMA particles (*k*_1_ = *k*_2_ = 2.6; *r*_1_ = *r*_2_ = 600 nm) suspended in hexadecane (*k*_*m*_ = 2.06) with added charge control agent (AOT) of different concentrations: AOT = 1 mM, *Q*_1_ = *Q*_2_ = 63*e* (a), and AOT = 10 mM, *Q*_1_ = *Q*_2_ = 60*e* (b). The force is also calculated using the DLVO theory and polarizable model^2^ with different values of the dielectric constant of interacting particles. Reprinted with permission from ref (2). Copyright 2016 American Institute of Physics.

Sainis et al.^33^ have performed optical trap measurements of the electrostatic force on PMMA spheres (*k* = 2.6) with a radius of 600 nm in hexadecane (*k*_*m*_ = 2.06) containing a soluble charge control agent of aerosol sodium di-2-ethylhexylsulfosuccinate (AOT). The electrostatic force was measured for molar concentrations of AOT corresponding to different values of particle charge and the Debye length of the medium. These measurements are presented in Figure 1 together with predictions of the DLVO theory and polarization model.^2^ It is evident that, at separation distances exceeding the Debye length, the experimental results are correctly described by both theoretical models. In the region of center–center separation between 1.2 μm (touching point) and 2 μm, which was not accessible to experimental measurements, polarization contributions to the electrostatic force are responsible for the small difference between the DLVO prediction and the polarizable electrostatic model.^2^ At the touching point, reduction in the magnitude of the electrostatic force due to polarization, relative to the DLVO theory, amounts to just 7%. In experiments by Sainis et al.,^33^ the difference between the values of the dielectric constant of the colloid particles and solvent is very small, and the magnitude of the charge on the particles is not high enough to cause significant polarization.

However, the marked difference in polarization of the particles and the medium will have a significant effect on their electrostatic interaction,^17^ leading to a large discrepancy between the DLVO theory and the more accurate solution.^2^ If the dielectric constant of particles in Sainis experiments^33^ was different from that of the solvent, the overall polarization effects would be much stronger, and the DLVO theory would provide less accurate results. To demonstrate this, two additional calculations have been added to Figure 1. The dash-dot line denotes the electrostatic force between two highly polarizable spheres (*k*_1_ = *k*_2_ = 80) of the same charge and size as colloidal particles in the experiment.^33^ In this case, polarization effects cause a considerable reduction in the magnitude of the electrostatic force, amounting at the point of contact to 37% difference with the DLVO predictions, which describe the interaction between point charges or not polarizable particles. The dash-double dot line in Figure 1 represents the case of not polarizable spheres (*k*_1_ = *k*_2_ = 1), for which the magnitude of the force at the contact point is 24% greater than the electrostatic force predicted by the DLVO theory. An increase in the magnitude of the repulsive force is caused by the polarizable medium. This discussion emphasizes the fact that, within the same chemical system, taking into account polarization effects can reveal different electrostatic behavior.

**Figure 2:**
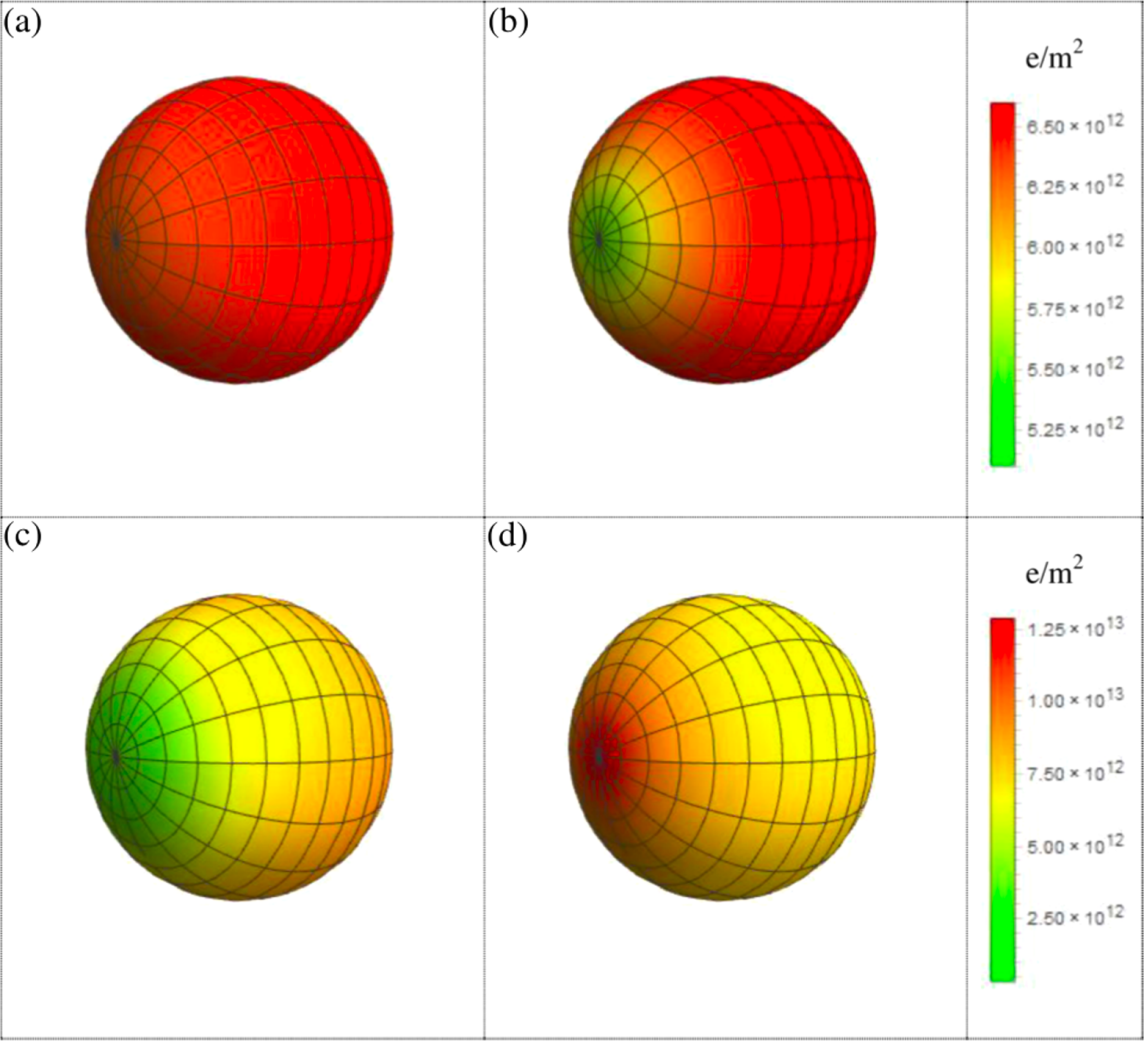
Total charge distribution on the surface of a 600 nm PMMA sphere suspended in hexadecane with 10 mM AOT: (a) *k*_1_ = *k*_2_ = 2.6 and center-to-center separation between the particles is equal to the Debye length; (b) *k*_1_ = *k*_2_ = 2.6 and the contact point; (c) *k*_1_ = *k*_2_ = 80 and the contact point; (d) *k*_1_ = *k*_2_ = 1 and the contact point. Reprinted with permission from ref (2). Copyright 2016 American Institute of Physics.

The calculations of the electrostatic force^2^ shown in Figure 1 are underpinned by the accurate predictions of the total surface charge. Examples of the calculated surface charge distributions are shown in Figure 2, which correspond to a 600 nm PMMA particle suspended in hexadecane with a 10 mM molar concentration of AOT, as used in Figure 1b. At separation distances comparable to the Debye length of the medium, the electrostatic interaction between the particles is shielded by the electrolyte, the surface charge distribution remains close to uniform (Figure 2a), and the DLVO theory remains to be reliable. Figure 2b depicts the highly nonuniform surface charge distribution, depleted at the point of contact due to surface charge polarization, thus explaining the reduction in the electrostatic repulsion between the particles described above. Other examples of strongly nonuniform distributions of the surface charge are shown in Figure 2: due to dominant polarization of the particles (Figure 2c) or due to dominant polarization of the medium (Figure 2d); note that the particle in Figure 2d is not polarizable. This discussion emphasizes the fact that, within the same chemical system, taking into account polarization effects can reveal different electrostatic behavior.

Generally, a rigorous model of the electrostatic problem in an electrolyte solution^2^ can be used more broadly to study colloidal systems with high and/or widely different dielectric constants and in a range of different solvent conditions including high concentrations of electrolyte. This, however, requires a detailed consideration of the boundary conditions of the problem.

### Selection of the Boundary Conditions

Models for electrostatic interactions in an electrolyte solution developed within the Debye–Hückel approximation, e.g. Derbenev et al.,^2^ often assume that free charge on a particle is constant and uniformly distributed over its surface. These models typically provide good agreement^34^ with predictions from nonshielded models^1,35^ and experimental measurements of electrostatic interactions in weak electrolytes.^33^ However, the assumption of constant surface charge density is not always valid, and in some chemical scenarios (typically, in colloid chemistry) the boundary condition of constant potential needs to be considered.^18^ In both cases, the surface of a particle represents the physical boundary which separates two different dielectric media, e.g., particle and solvent (or vacuum), leading to a discontinuity in the electric and dielectric displacement fields.

**Figure 3:**
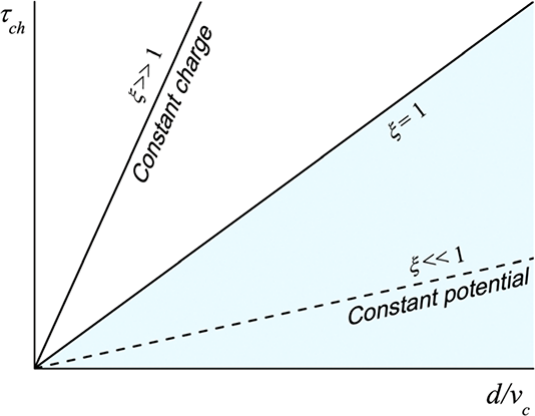
Selection of an appropriate boundary condition (constant charge or constant potential) by comparing the characteristic time of particle charging, τ_*ch*_, and the characteristic time of particle displacement, *d*/*v*_*c*_. Reprinted with permission from ref (18). Copyright 2018 Royal Society of Chemistry.

For selecting an appropriate boundary condition, we introduce a simple dimensionless parameter
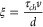
2where τ_*ch*_ is the relaxation time of the surface charge, *v* is velocity of the particles, and *d* is the characteristic surface-to-surface separation between the particles over which the surface charge or surface potential can change.^18^ If the particles are stationary (*v* = 0) or the time scale of their interaction is much greater than the characteristic relaxation time of the surface charge (τ_*ch*_ ≪ 1) then ξ ≪ 1 and the boundary condition of constant potential is applied. Practically, this condition is suitable for the cases where particles are much larger than the Debye length or if the concentration of the electrolyte is high. If the particle charging process is much slower than the time it takes for the particle to travel the distance equivalent to its size, then ξ ≫ 1 and the surface charge is taken to be constant. Figure 3 depicts the relationship between the characteristic time of particle charging and that of particle displacement for different values of ξ; this can be a helpful guide for selecting the boundary conditions when solving the problem of electrostatic interaction between charged particles.

The velocity in eq 2 depends on the particle motion, either ballistic or diffusive, which is defined by the mean free path and the characteristic length of variation in the surface charge or potential. In the ballistic regime, the mean free path of a particle is typically much greater than the particle size (as defined by its radius, *a*). If *v* represents the velocity of the thermal motion then the parameter ξ can be expressed as^18^
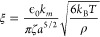
3where ρ is the density of the particle, ζ = *e*(μ_*p*_*n*_*p*_ + μ_*n*_*n*_*n*_) is the conductivity of the solution, and μ_*p*_, *n*_*p*_, μ_*n*_, *n*_*n*_ are the mobility and concentration of positive and negative ions. In the diffusive regime, when the mean free path of a particle is significantly less than the particle’s size, the parameter ξ takes the form^18^
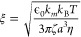
4where η is the dynamic viscosity of the medium.

Equations 3 and 4 complete a simple selection criterion for the choice of the boundary conditions in the electrostatic problem solved through the use of the Poisson–Boltzmann equation to obtain the electrostatic force (or energy) as an infinite series of the charge-induced multipolar terms. This selection criterion depends only on the physical properties of the interacting particles and the interaction conditions. It gives clear, intuitive guidance to computations based on available experimental data such as the conductivity and viscosity of a solution and the dimensions and density of particles.

One observation stemming from eqs 3 and 4 is that the boundary condition of constant charge (ξ ≫ 1) can be realized only for particles in the submicrometer size range (*a* ≪ 1 μm) or for dilute solutions, typically, less than 10^–2^ mM. For larger particles, it is important to compare their size with the value of the mean free path. For example, the mean free path of colloidal particles in water is λ_*c*_ ≈ 5 × 10^–10^ m and λ_*c*_ ≈ 10^–10^ m in hexadecane, which are both much smaller than the particle size of an ∼1 μm. These are examples of a diffusive motion which is typical for electrolyte solutions, while the ballistic motion is often observed in dusty plasmas. Therefore, the Derbenev et al. model^2^ based on the Debye–Hückel approximation can be used to describe electrostatic interactions in an electrolyte solution, dusty plasmas, and other complex scenarios involving charged particles in a neutralizing environment.

**Figure 4:**
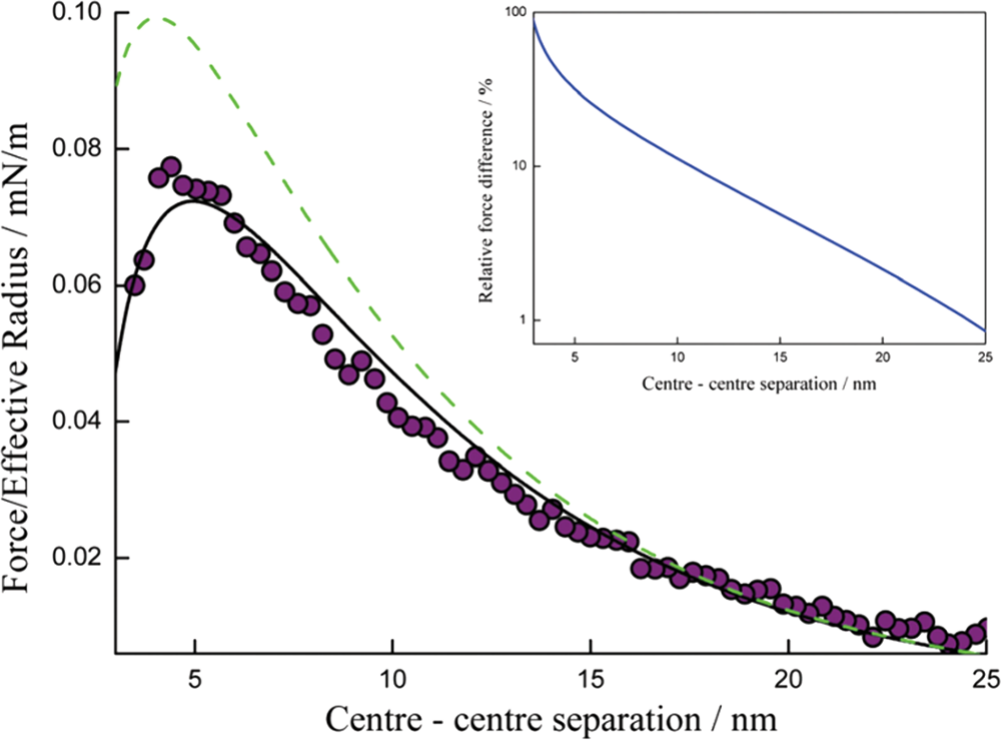
Force between two latex charged particles of radius 0.97 μm in electrolyte solution at 3.0 pH and 1 mM KCl concentration: filled circles are the experimental data,^36^ solid line is the force calculated using the methodology,^18^ dashed line is the DLVO prediction using the same parameters of κ^–1^ = 6.9 nm and Φ_surface_ = 14 mV. In the inset, the relative difference (in %) between the exact and approximate DLVO force. Reprinted with permission from ref (18). Copyright 2018 Royal Society of Chemistry.

An excellent example highlighting the requirement for the use of constant potential boundary condition is found in the experimental measurements of Montes Ruiz-Cabello et al.^36^ where the electrostatic force between charged latex particles has been recorded at different pH values and KCl salt concentrations for particles of different sizes. Figure 4 presents experimental data recorded for a particle radius of 0.97 μm. The surface potential in this case is Φ_surface_ = 14 mV, which is below the thermal energy and equates to the zeta-potential.^36^ For the given experimental conditions, i.e. KCl concentration of 1 mM at pH = 3.0 and *T* = 298.25 K, the Debye length is approximately 6.9 nm.^18^ Application of the ξ selection criterion reveals that, at the considered salt concentration of 1 mM, the constant potential boundary condition is appropriate if the particle radius exceeds 0.1 μm. The electrostatic force, calculated using the methodology,^18^ has been supplemented with the van der Waals force using the Derjaguin approximation.^37^ The comparison of experimental and theoretical results presented in Figure 4 show that the discrepancy between the quantitatively accurate force^18^ and its approximation from the DLVO theory can reach 100% at close separation, and a difference of about 10% begins to accumulate at the separation distances of 2–3 D lengths.

## Theory of Many-Body Electrostatic Interactions

### Beyond the Image Charge Model

Calculations of electrostatic interactions are often based on the image charge model, which was proposed in 1845 by Thomson, later Lord Kelvin, to solve the problem of a point charge located outside a conducting sphere kept at zero potential. Thomson used the Legendre polynomials to express the electric potential due to the actual charge as the potential due to an imaginary point charge.^38^ In the late 1990s, the classical Kelvin image theory for a charged sphere was extended by Lindell^39,40^ to include conducting objects of other shapes, such as prolate spheroids, and dielectric spheres.^41−43^ Numerous extensions of the image charge theory are suitable for describing many aspects of experiment.

Following the ideas of the mean-field theory, Freed^44^ used a local expansion of the many-body electrostatic problem in order to reduce it to a one-body problem. They described the effect of the electric field induced on a particle by all other particles present in the system and solved the one-body electrostatic problem iteratively and self-consistently to achieve the desired convergence. This approach reduces computational cost, but the adopted iterative procedure has inherent convergence problems, especially at short separations. A mathematically more rigorous approach is to start with a global many-body formulation of the problem and interpret the many-body expansions as a block-Jacobi iteration scheme, where each block corresponds to one particle.

A desirable feature of a useful electrostatic model is its ability to relate the computed interactions between charged particles to the origin of the predicted electrostatic behavior rooted in an accurate account of the surface charge distribution on each particle. In the many-body setup at short separation distances, the surface charge distribution can be very complex and is unique for every geometry and separation distance. A quantitatively accurate account of the surface charge can explain many fragmentation and assembly processes, which cannot be captured by more approximate solutions. Both in static and dynamics simulations, the instantaneous redistribution of surface charge can be calculated numerically, e.g. using the Galerkin method and its derivatives,^4,16^ or analytically.^3^ It is not possible, however, to extract these insights from the image charge model or mean-field methods.

Multipole expansion approaches^4,17,45^ have an advantage of providing physical insight into the electrostatic nature of the interactions through the predictions of how the surface charge behaviors. Often, analytical expressions for the surface charge distribution are quite simple, thus allowing for further testing of the electrostatic solutions against accurate quantum chemical methods such as density functional theory.^46,47^

### Numerical Realizations

An accurate description of electrostatic interactions using multipole expansion methods can become prohibitive in terms of computation time if the geometry of a chemical system requires the use of a large number of multipole terms. Description of electrostatic interactions in many-body dielectric systems is an intrinsically complex problem as the total charge distribution on the interfaces, namely, surfaces of all interacting particles and supports, is a result of the coupled polarization effects taking place in the particles and medium. In the many-body setup, each pair interaction cannot be calculated independently and symmetry constraints, which can be redily imposed on a two-body system, are not applied to three or more particles. The interactions of several dielectric particles can be described by a generalized Poisson equation, which is often reduced to a boundary integral equation (BIE) representing the induced surface charge on the particles. Numerical methods, such as the Boundary Element Method (BEM)^48,49^ or the Method of Moments (MoM),^45,50^ can be viewed as a discretization of a BIE.

Nevertheless, for a many-body system it is important to provide a rigorous characterization and mathematical framework of the exact solution, which contains no discretization errors. This was achieved by Lindgren et al.^16^ in a well-founded mathematical approach using a variational formulation of the problem in terms of a BIE of the second kind and a spectral Galerkin approximation. Furthermore, no errors were introduced in approximating the geometry of the problem as no meshing is required, leading to an efficient discretization of polydisperse configurations. The mathematical formalism^16^ combines variational aspects of the BEM-based solutions with the high order character of the MoM. Both the continuous solution^16^ and the Galerkin approximation are well characterized and offer a rigorous convergence of the induced polarization surface charge, the electrostatic interaction energy, and the net forces acting on particles. The many-body description,^16^ based on the second kind BIE, expands on earlier works, for example, by Juffer et al., who presented a boundary element method to compute the electric potential for a single macromolecule in a solvent with given ionic strength,^51^ and later extended this method to describe ionic strength by means of explicit ions for the case of two polarizable regions.^52^

The solution^16^ can be applied to a large number of particles of arbitrary size, charge, position and dielectric constant, embedded in a homogeneous medium. The computation using the derived algorithm scales linearly with respect to the number of particles in the system, and the rigorous tests of the convergence, timing, and linear scaling can be found in section 3.1 of ref (16). The linear scaling of the problem has been achieved through the use of a modified fast multipole method (FMM) by noting that a surface charge represented by a truncated series of spherical harmonics and a corresponding multipole located at the center of a sphere representing each particle can be treated as equivalent. Generally, the complexity of the underlying expansions scales with the fourth power of the degree of spherical harmonics and can be reduced to the third power with more efficient FMM-embedding. This gives the same asymptotic scaling as a hybrid method proposed by Gan.^53,54^ In some very specific cases, when azimuthal symmetry can be assumed, quadratic scaling with the degree of spherical harmonics can be achieved.

## Experimental Cases

An integral equation approach,^16^ which through the use of FMM method scales linearly with respect to the number of particles, has been combined with a simple solution of the classical equations of motion to predict time evolution of charged particle assembly processes (classical dynamics).^3^ In the following, we discuss two experiments where electrostatic assembly has been promoted in the absence of additional constraints, such as solvent or ionic medium.

Whitesides and co-workers^55−57^ used contact electrification to create two-dimensional models of electrostatic self-assembly. Millimeter-size polymer spheres of varying size and composition, nylon, Teflon, etc. have been subjected to tribocharging to accumulate either a positive or negative charge on the order of a few hundred picocoulombs (pC). Electrostatically driven self-assembly of these charged particles into different two-dimensional lattice structures has been recorded, with the resultant lattice motifs varying according to particle charge, size, and the fraction of each polymer type. Many-body simulations^3^ explored an extensive range of the experimental parameter set including the effects of particle charge, dielectric constant, and the ratio of the number of negatively and positively charged particles.

**Figure 5:**
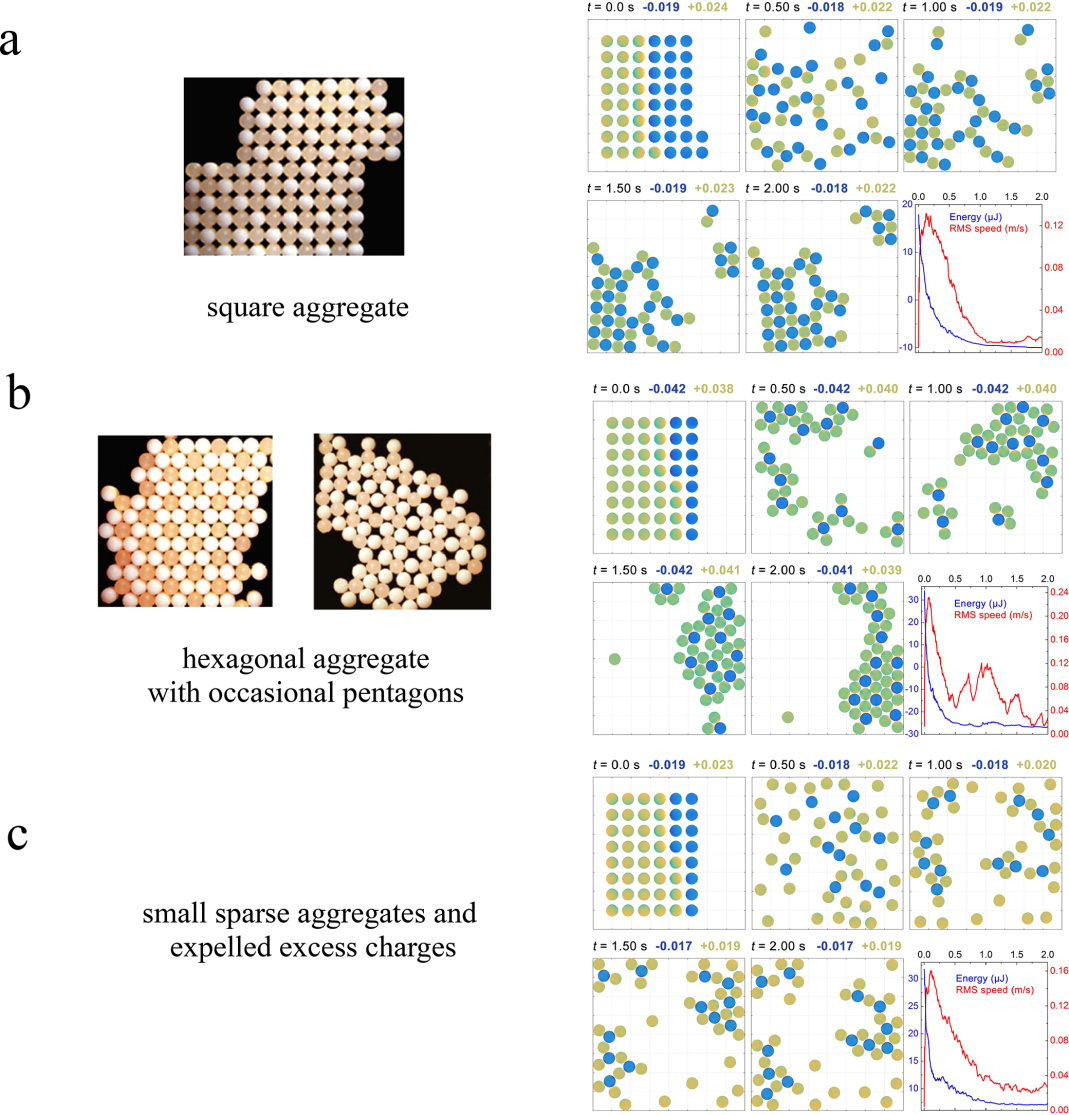
Particle dynamic simulation (right) of an experiment by Whitesides and co-workers^55^ (left). The numbers at the top of each panel are the maximum calculated surface charge density in nC mm^–2^. The green/yellow spheres represent PMMA particles (*d* = 1.59 mm, *k* = 3.2), and the blue spheres represent Teflon particles (*d* = 1.59 mm, *k* = 2.1). (a) PMMA/Teflon particle number ratio 1:1, *q*_PMMA_ = +0.31 nC and *q*_Teflon_ = −0.31 nC (neutral aggregate); (b) PMMA/Teflon ratio 3:1, *q*_PMMA_ = +0.31 nC and *q*_Teflon_ = −0.93 nC (neutral aggregate); (c) PMMA/Teflon ratio 3:1, *q*_PMMA_ = +0.31 nC and *q*_Teflon_ = −0.31 nC (charged aggregate). The time evolution of the interaction energy, in μJ, and the RMS velocity of the particles, in m s^–1^, are also included. Reprinted with permission from ref (3). Copyright 2018 Royal Society. Adapted from ref (55) with permission from Springer Nature.

Figure 5 summarizes the results of particle dynamic simulations showing how collections of poly(methyl methacrylate) PMMA/Teflon particles assemble on a surface into a range of 2D lattice structures depending on the composition and charge state of the particles. If the PMMA/Teflon number ratio is 1:1 and the overall system is charge neutral, square arrangements of alternating PMMA and Teflon particles are formed (Figure 5a); for a PMMA/Teflon ratio of 3:1 and a neutral system, hexagonal aggregates with the occasional presence of pentagons are observed (Figure 5b), and finally, small sparse aggregates and expelled excess charges are observed for a PMMA/Teflon ratio of 3:1 and an overall unbalanced charge in the system (Figure 5c). This is very similar to experimental observations.^55−57^ From these simulations, it can be concluded that the resultant lattice structures are sensitive to the proportion of negatively and positively charged particles present in the collection and to the amount of charge on each particle.

Another experimental example by Lee et al.^58^ captured images of submillimetre-size particles clustering in charged granular streams. Time sequences of falling particles in a vacuum under gravitational force displayed orbital binary collisions and clustering events. These experiments differ from those of Whitesides and co-workers in several ways: first, the particles were smaller (approximately 300 μm) and carried less charge (approximately 0.1 pC), but more significantly, the material used, a composite of zirconium dioxide and silicate, had a much higher dielectric constant (*k* = 15) than any of the polymer spheres used by Whitesides and co-workers (*k* varies between 2 and 4).

Particle dynamics simulations based on the many-body polarization electrostatic model^3^ reproduced the key collision processes observed in Lee et al. experiments,^58^ which include (1) capture of an individual particle by a small cluster, when the center-of-mass collision energy is lower than the binding energy of the particle to the cluster or if it is effectively dissipated through the cluster; (2) particle escape showing an incoming particle bouncing away from a stable cluster; and (3) cluster fragmentation seen at high collision velocities leading to a complete breakup of a cluster.

## Larger Scope for Applications and Conclusions

The theories developed can be used not only to understand and explain experimental findings but also to predict and discover new phenomena. Lindrgen et al.^17^ investigated how the properties of a solvent could either facilitate or suppress electrostatic fabrication, based on experiments^59^ involving the interaction of neutral alumina nanoparticles with a charged nanodiamond surface. Experiments^59^ show that neutral alumina particles will be attracted to the surface if they are immersed in a low dielectric medium, i.e. insulating fluorocarbon solution, fluorinert FC-90. Further calculations^17^ predict that the nature of this electrostatic interaction can switch markedly from being attractive to repulsive in a solvent with higher dielectric constant, for example, acetone.

There are several examples in the literature of deposition processes pointing at the evidence of a critical charge density being required for the assembly and growth of thin films.^60^ For example, most polyoxometalate clusters (POMs) are soluble in water but it is widely recognized that it is not possible to fabricate POMs layers from such polar medium without first creating a charged substrate.^61^ Extensive calculations^17^ have been undertaken to model the deposition of the Eu-POM onto a layer of positively charged macromolecules and to describe experimental conditions required for the successful electrostatic self-assembly. These calculations generally explore the consequences of charged particles interacting in a wide range of solvents covering interactions between both opposite- and like-charged particles and size ratios that span from particles of equal size to significantly different.

Materials research poses many additional computational challenges, which require fundamental analytical solutions capable of quantitatively accurate descriptions of electrostatic interactions and the interpretation of particle assembly and fragmentation. External stimuli, such as applied electric fields, often drive the assembly of particles into new functional forms, with electromagnetic radiation, localized surface charges, templating on supporting substrates, different aspect ratio of building blocks (sphere, rod, wire, disc, ...), adding complexity to method development.

The formalism^16^ was extended further by Hassan et al.^4^ to include the interactions of a many-body system with an external electric field and in the presence of localized (point) charge on the particle surface. These new computational features add significant complexity to the mathematical model due to the nondecaying character of an external electric potential that does not vanish at infinity and due to the presence of singularities arising in the context of the surface point charge. However, incorporating these important effects into the existing methodology considerably broadens its applicability and provides a versatile method for studying many important physical, chemical, and industrial processes previously inaccessible to accurate computation.

For example, the proposed method^4^ has been applied to study the stability and melting of ionic colloidal crystals in an external electric field. Leunissen et al.^22^ investigated the formation of apolar colloidal crystals consisting of PMMA particles of different size and opposite and dissimilar charges suspended in a density matching mixture of cyclohexyl bromide (CHB) and *cis*-decalin. For a wide range of particle sizes and charges, body-centered cubic-type (cesium chloride) crystals were formed, which could be reversibly destabilized by the application of an electric field. This behavior was explained by calculating the electrostatic force acting on charged particles in an external electric field.^4^ The force acting in the direction of the applied field creates a surface charge distribution different from that in the absence of the field. When exposed to a sufficiently high electrical field, the resultant surface charge distribution leads to repulsion between particles in the plane perpendicular to the direction of the field.

The many-body formalism^16^ has been further used to identify nanoparticle lattices and endohedral fullerenes as potential building blocks for future electronic, magnetic and optical devices. For example, Miller et al.^21^ proposed that it could be possible to design stable nanoparticle lattices composed from binary collections of endohedral fullerenes.

In conclusion, comprehensive electrostatic theories^1,2,4,5,9,14−16,18^ have been developed, rigorously tested and widely applied to accurately describe and explain fragmentation and coalescence processes, where induced surface charge polarization plays a critical role at short separation distances—the region where previous approximate solutions failed to provide accurate results. The methods developed have been used to analyze electrostatic effects in a diverse range of applications including, but not limited to, dusty plasmas and planetary environments^2,11,12,18^ Coulomb fission in multiply charged clusters^6−9^ and in soft matter,^13^ including a counterintuitive but widespread phenomenon of attraction between like-charged polarizable particles.^10^ This continued interest in new electrostatic solutions is also motivated by emerging self-assembly processes and packing of nanomaterials,^21^ often directed and controlled by external fields,^4^ templates, or directing agents.
